# Pelvic Sepsis Secondary to Necrotic Coloanal Anastomosis After Perineal Proctosigmoidectomy Leading to Emergent Abdominoperineal Resection: A Rare Case and Complication

**DOI:** 10.7759/cureus.98821

**Published:** 2025-12-09

**Authors:** Prathusha Yerramilli, Michael Boysen, Kristina Fioretti, Stevie-Jay Stapler

**Affiliations:** 1 Surgery, Trinity Health Ann Arbor Hospital, Ypsilanti, USA; 2 General Surgery, Trinity Health Ann Arbor Hospital, Ypsilanti, USA; 3 Colorectal Surgery, Trinity Health Ann Arbor Hospital, Ypsilanti, USA

**Keywords:** abdominoperineal resection (apr), altemeier procedure, pelvic sepsis, perineal proctosigmoidectomy, robotic colorectal surgery

## Abstract

Abdominoperineal resection (APR) is a surgical procedure that involves the complete removal of the anal canal, anal sphincter complex, rectum, and a portion of the sigmoid colon with creation of an end colostomy. This procedure is primarily indicated for patients with low rectal cancers where the anal sphincters cannot be spared and anal cancers refractory to chemoradiation or recurrent anal cancers. It can also be performed in patients with severe perianal Crohn’s disease, complex anorectal fistulae, or patients with severe trauma to the anus/rectum where the sphincter complex is significantly affected. This is a complex procedure with a high complication rate, and patients generally undergo extensive perioperative planning and assessment to optimize a positive outcome. In this report, we describe a rare case of emergent APR in a patient who presented with pelvic sepsis secondary to necrotic rectum and colon after a prior emergent Altemeier procedure at another hospital. A thorough literature review has revealed only one prior documented case of emergent APR.

## Introduction

Incarcerated rectal prolapse is a severe form of full-thickness rectal prolapse in which the prolapsed segment becomes irreducible, leading to venous congestion, edema, and potential strangulation. This condition occurs most commonly in elderly or frail patients with chronic constipation, weakened pelvic floor musculature, and comorbidities that impair tissue integrity. When blood flow becomes compromised, the prolapsed rectum is at risk of ischemia, necrosis, or even perforation, which can rapidly progress to pelvic sepsis if not addressed. Consequently, prompt surgical intervention is required, particularly in elderly or medically fragile patients who are at greatest risk for these life-threatening complications [1].

Perineal proctosigmoidectomy, also known as the Altemeier procedure, is a relatively safe and effective option for full-thickness rectal prolapse, especially in frail or elderly patients for whom an abdominal approach may be contraindicated. It can be performed under regional anesthesia, is associated with low postoperative morbidity, minimal pain, and a short recovery period. However, its principal limitation is a non-negligible recurrence rate and potential complications, which include anastomotic dehiscence, pelvic hematoma, abscess formation, and anal stricture. Overall, the Altemeier procedure remains a valuable surgical alternative for medically fragile patients, but it is not without risk, particularly in cases of incarcerated prolapse where vascular compromise, poor tissue quality, or subsequent interventions such as arterial embolization can predispose to anastomotic failure and pelvic sepsis [2]. Such complications can require complex surgical intervention to manage, as demonstrated in the present case, where the patient underwent emergent abdominoperineal resection (APR). 

APR is a surgical procedure that involves the complete removal of the anal canal, anal sphincter complex, rectum, and a portion of the sigmoid colon. Patients are thus left with a permanent end colostomy (usually end descending colostomy) to divert stool. This procedure is primarily indicated for patients with low rectal cancers where the anal sphincters cannot be spared and anal cancers refractory to chemoradiation or recurrent anal cancers. It can also be performed in patients with severe perianal Crohn’s disease, complex anorectal fistulae, or patients with severe trauma to the anus/rectum where the sphincter complex is significantly affected [3]. 

APR is a technically complex operation due to the inherent challenges of operating in the pelvis, including risk of hemorrhage from the presacral venous plexus and the risk of injuring neighboring structures, including the vagina, prostate, ureters, and pelvic nerves. It is well described in the literature to be associated with a high rate of complications, including blood loss anemia requiring transfusion, wound infection, perineal wound dehiscence, intra-abdominal abscesses, postoperative ileus, ureteral injury, sexual or urinary dysfunction due to autonomic nerve injuries, and stoma-related complications [3-4]. 

To ensure the best outcomes for patients in this complex procedure, ideally, patients would have preoperative nutrition optimization and counselling via an enhanced recovery after surgery (ERAS) pathway to ensure appropriate mechanical and antibiotic bowel preparation, preoperative carbohydrate loading, pre- and postoperative opioid receptor blockers such as alvimopan, and fluid restriction intraoperatively to target euvolemia [3]. 

In short, this is an operation that, in an ideal scenario, would be scheduled electively and include a multidisciplinary approach, emphasizing preoperative assessment and patient preparation to optimize patient outcome and reduce morbidity. It is extremely rare to undertake APR in the emergent setting; after a thorough literature review, only one prior documented case could be found [5].

## Case presentation

The patient was an 80-year-old male with a history of atrial fibrillation on apixaban, coronary artery disease status post bypass grafting, hypertension, and rectal prolapse for which he deferred surgical management, who initially presented to the emergency department (ED) of another facility with incarcerated prolapse. He was taken to the operating room (OR) for an emergent Altemeier procedure and recovered from it appropriately. He was discharged on postoperative day 4. He returned to that facility multiple additional times for urosepsis and persistent hematochezia requiring cessation of his anticoagulation. During his last admission, about 3 weeks after the index operation, he presented with urosepsis as well as copious hematochezia. He was hypotensive and in septic vs. hemorrhagic shock. Ultimately, he underwent embolization of his superior rectal artery with interventional radiology (IR), which controlled his bleeding. His urosepsis was managed with antibiotic therapy, and he was discharged to a rehabilitation facility. 

**Figure 1:**
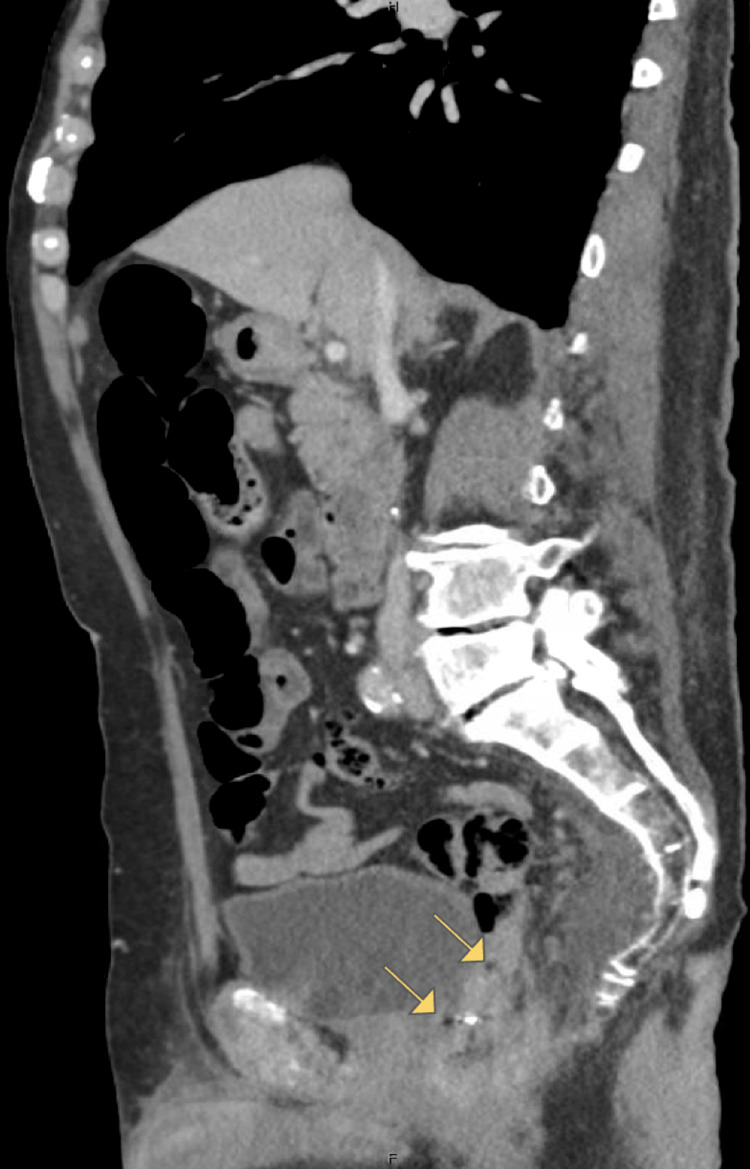
CT imaging showing heterogenous perianal fluid collection and segmental mural thickening of the rectum, as well as spots of intraperitoneal air (arrows) adjacent to the anastomotic staple line. CT = computed tomography

Two weeks later, he presented to the ED with an 11-day history of copious bloody and purulent drainage from the anus, lethargy, and tachycardia. Computed tomography (CT) imaging on presentation revealed a heterogeneous perianal fluid collection and segmental mural thickening of the distal rectum and left aspect of the anal canal (Figure 1). Furthermore, the sagittal views of the CT showed concern for small volume free air just proximal to the coloanal anastomosis (Figure 1). Bedside anoscopy and digital rectal examination (DRE) were notable for sanguinopurulent rectal drainage, as well as a palpable defect at the coloanal anastomosis, concerning for at least 50% dehiscence or more. Additionally, necrotic mucosa was visualized distally. The purulent discharge made further visualization quite difficult. As he was febrile and tachycardic, he was promptly taken to the OR for an examination under anesthesia (EUA) given concern for ischemia. 

**Figure 2:**
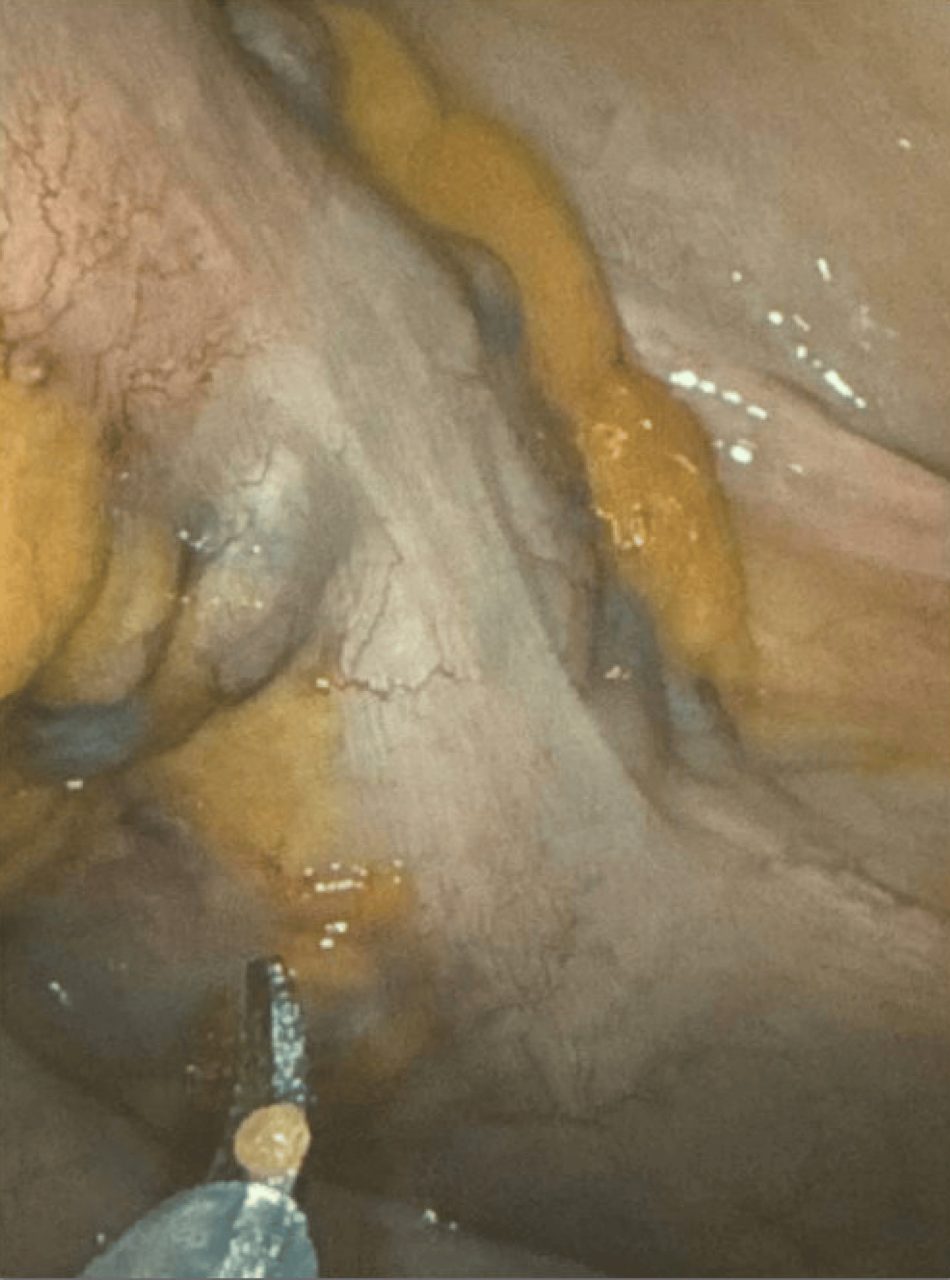
Intraoperative image showing demarcation between pale and ischemic distal colon/rectum compared to healthy perfused upstream colon

Examination under anesthesia confirmed anastomotic dehiscence with findings of a large defect along the entirety of the posterior aspect of the coloanal anastomosis. The necrotic appearing mucosal changes extended more proximally than what could be visualized on EUA alone, so a flexible sigmoidoscopy was also performed. This was notable for a posterior defect that appeared to be approximately 75% of the circumference of the coloanal anastomosis. Furthermore, seropurulent fluid and stool were present in the cavity that was overlying the distal sacrum and coccyx. We then performed diagnostic laparoscopy to visualize any transmural inflammation. The distal colon and rectum were pale in appearance and confirmed to be ischemic with no uptake of indocyanine green (Figure 2). Given these findings, it was apparent that the patient would need resection with colostomy. As the patient had no clearly viable portion of his anal canal that could hold a suture line or staple line, the decision was made to undertake emergent robotic abdominoperineal resection with end colostomy. Patient’s hemodynamics were able to tolerate insufflation prior to initiation, and the robotic approach was selected due to the efficiency of pelvic dissection and optimal visualization to decrease operative time, as well as surgeon preference. 

His postoperative course was complicated by ileus requiring nasogastric tube (NGT) placement twice, and he was placed on total parenteral nutrition (TPN) support for a total of 12 days. Ultimately, NGT was removed, and he was able to tolerate sufficient oral intake to maintain nutritional needs. His course was also complicated by an intra-abdominal abscess requiring interventional radiology drain placement and dehiscence of the perineal wound at the skin, requiring wound vac placement. He was ultimately discharged home on postoperative day 17 with home nursing to assist with new ostomy care and wound vac changes. 

The patient was seen in the outpatient colorectal surgery clinic for routine postoperative and wound care appointments 8 days after discharge, and was doing well. He was tolerating diet without issue, and his stoma was functional. Furthermore, his perineal wound was healing appropriately. He has since had weekly monitoring of his wound, and no further concerns were noted.

## Discussion

Our patient initially underwent an Altemeier procedure for incarcerated rectal prolapse, complicated by recurrent lower GI bleed necessitating IR embolization. It is possible that this embolization compromised blood flow to his anastomosis, which contributed to his presentation with pelvic sepsis from acutely necrotic rectum and colon.

The colon has significantly fewer collateral channels for blood flow than the small bowel, and is thus at a higher risk for ischemic insult if the main blood flow is compromised [6]. Blood flow to the patient’s coloanal anastomosis would have originated from the inferior mesenteric artery (IMA). Since the superior hemorrhoidal artery was embolized during the IR procedure, it could explain the necrosis seen intraoperatively. Furthermore, the patient had been experiencing persistent rectal bleeding and malaise, leading to poor oral intake prior to presentation. He had been hypotensive in the ED, and it is unknown how long this hypotension had been ongoing prior to presentation. This low-flow state would have further contributed to poor colonic perfusion. 

The primary blood supply following an Altemeier procedure has not been well studied. However, it is reasonable to suggest that the collateral flow of the native rectum is disrupted during this operation, amplifying any further insults to perfusion, as in the case of our patient. Our primary takeaway from this case is to be very judicious about the management of anastomotic bleeding following a perineal proctectomy. Operative exploration is always a reasonable approach, and given this complication, in the future, it could be prudent to discuss resection with end colostomy as another treatment option. Alternatively, it would be appropriate to discuss the possibility of anastomotic complications if embolization is considered following an Altemeier. 

When blood flow to the colon is compromised, it can become necrotic, which can lead to thinning of the bowel wall and eventual perforation, with spillage of stool into the abdomino-pelvic cavity. New anastomoses, such as those our patient had from his perineal proctosigmoidectomy, are especially at risk for dehiscence with ischemic insult as they are still healing. The feared severe complication from this scenario would be pelvic sepsis secondary to stool spillage, which our patient did experience. Pelvic sepsis is characterized by fever, abdominal pain, and leukocytosis and can progress quickly into septic shock, characterized by the aforementioned symptoms as well as refractory hypotension and end-organ damage. In patients with pelvic sepsis, or sepsis of any kind, obtaining source control is important in order to keep the patient from progressing to death. 

Our patient presented with persistent rectal bleeding, fever, abdominal pain, urinary retention, and hypotension, concerning for pelvic sepsis. CT imaging showed an irregular, thickened appearance of the distal rectum and left aspect of the anal canal. Furthermore, the sagittal views of the CT showed concern for small volume free air just proximal to the coloanal anastomosis. Given his symptoms and hypotension coupled with DRE concerning for a palpable defect in the anastomosis, he was taken to the OR, where exploration showed a large defect that appeared to be approximately 75% of the circumference of his anastomosis. Furthermore, seropurulent fluid and stool were present in the cavity that was overlying the distal sacrum and coccyx, and on exam, the patient had no clearly viable portion of his anal canal that could hold a suture line or staple line. This was further confirmed with an indocyanine green injection showing no uptake in the distal colon and rectum. He thus required emergent APR. Despite the fact that APR is a procedure that is usually not performed in the emergent setting, it was the only way to gain source control and treat this patient’s pelvic sepsis.

## Conclusions

This case report highlights the extremely rare occurrence of emergent APR. In summary, APR is a surgical procedure that involves the complete removal of the anal canal, anal sphincter complex, rectum, and a portion of the sigmoid colon with creation of an end colostomy. This is a complex procedure with a high associated morbidity, and patients generally undergo extensive perioperative planning and assessment to optimize a positive outcome. APR is almost never done in the emergent setting. Our patient initially underwent an Altemeier procedure for rectal incarceration, which was complicated by recurrent lower GI bleed necessitating IR embolization. It is very likely that this embolization compromised blood flow to his coloanal anastomosis, which led to his presentation with pelvic sepsis from acutely necrotic rectum and colon. In this case, emergent APR was necessary to resect the necrotic bowel and obtain source control to treat pelvic sepsis.
